# Sertraline concentrations in pregnant women are steady and the drug transfer to their infants is low

**DOI:** 10.1007/s00228-021-03122-z

**Published:** 2021-03-22

**Authors:** E. Heinonen, M. Blennow, M. Blomdahl-Wetterholm, M. Hovstadius, J. Nasiell, A. Pohanka, L. L. Gustafsson, K. Wide

**Affiliations:** 1 Division of Paediatrics at the Department of Clinical Science, Intervention and Technology (CLINTEC), Karolinska Institute, Stockholm, Sweden; 2 Department of Paediatrics and Newborn Medicine, Karolinska University Hospital, Stockholm, Sweden; 3 Psychiatry South West, Stockholm Healthcare Region, Stockholm, Sweden; 4 Department of Clinical Sciences, Danderyd Hospital, Karolinska Institute, Stockholm, Sweden; 5 Department of Obstetrics and Gynaecology, Danderyd Hospital, Stockholm, Sweden; 6 Division of Clinical Pharmacology at the Department of Laboratory Medicine, Karolinska Institute, Stockholm, Sweden; 7 Department of Clinical Pharmacology, Karolinska University Hospital, Stockholm, Sweden; 8 Department of Paediatrics and Emergency Paediatrics, Karolinska University Hospital, Stockholm, Sweden

**Keywords:** Antenatal depression, Infant, Pharmacokinetics, Pregnancy, Selective serotonin reuptake inhibitors, Therapeutic drug monitoring

## Abstract

**Purpose:**

Sertraline, a selective serotonin reuptake inhibitor (SSRI), is one of the most commonly used antidepressant during pregnancy. Plasma sertraline concentrations vary markedly between individuals, partly explained by variability in hepatic drug metabolizing cytochrome P450-enzyme activity. Our purpose was to study the variability in the plasma concentrations in pregnant women and the passage to their infants.

**Method:**

Pregnant women with moderate untreated depression were recruited in 2016–2019 in Stockholm Region and randomized to treatment with sertraline or placebo. All received Internet-based cognitive behavior therapy as non-medical treatment. Sertraline plasma concentrations were measured around pregnancy weeks 21 and 30, at delivery, 1-month postpartum, in cord blood and at 48 h of age in the infant. The clinical course of the infants was followed.

**Results:**

Nine mothers and 7 infants were included in the analysis. Median dose-adjusted sertraline concentration in second trimester was 0.15(ng/mL) /(mg/day), in third trimester and at delivery 0.19 and 1-month postpartum 0.25, with a 67% relative difference between second trimester and postpartum. The interindividual variation was 10-fold. Median concentrations in the infants were 33% and 25% of their mothers’, measured in cord blood, and infant plasma, respectively. Only mild and transient adverse effects were seen on the infants.

**Conclusion:**

Placental passage of sertraline to the infant is low. However, the interindividual variation in maternal concentrations during pregnancy is huge, why therapeutic drug monitoring might assist in finding the poor metabolizers at risk for adversity and increase the safety of the treatment.

**Trial registration:**

The trial was registered at clinicaltrials.gov July 9, 2014 with TRN: NCT02185547.

**Supplementary Information:**

The online version contains supplementary material available at 10.1007/s00228-021-03122-z.

## Introduction

Sertraline is one of the most commonly used selective serotonin reuptake inhibitors (SSRI) during pregnancy, and generally proven safe for this use [1–6]. Plasma sertraline concentrations are shown to both increase and decrease during pregnancy [7–9]. No clear association has been found between the dose of sertraline or the plasma sertraline concentration and the clinical effect [10–15]. Plasma sertraline concentrations seem to vary up to 10-fold between individuals, but with a low intraindividual variation, with a recommended therapeutic range of 10–50 ng/mL [12, 16, 17]. The inter- and intraindividual variations seem similar in pregnant and non-pregnant populations.

Sertraline and desmethylsertraline are weak basic compounds. Sertraline is over 98% protein bound in plasma, binding to both albumin and alfa 1-acid-glycoprotein (AAP), but most likely mainly to AAP [18–20]. The levels of both albumin and AAP decrease during pregnancy, potentially affecting the sertraline plasma concentrations [21]. Multiple Cytochrome P450 (CYP) enzymes in the liver metabolize sertraline to its main weakly active metabolite N-desmethylsertraline [22]. The activity of these enzymes is genetically coded [23]. Current consensus is that CYP2C19 has a major role in sertraline metabolism and that the genetic differences in its expression cause interindividual variability in sertraline concentrations. The importance of CYP2B6, CYP3A4, CYP2C9, and CYP2D6 for the disposition of sertraline in vivo is not yet clear [22, 24–26]. Pregnancy induces changes in metabolic enzyme activity, with the activity of CYP2C19 being slightly down-regulated, and the activities of the other potentially important enzymes being up-regulated [7–9, 21]. As the pharmacokinetics change during pregnancy, therapeutic drug monitoring (TDM) might be a way to improve both safety and efficacy of the treatment [7, 12, 16, 17].

The infant albumin level at birth is slightly higher than the maternal, whereas the level of AAP is only a third of the mothers [27]. In previous studies, the placental penetration of sertraline to infant serum has seemed low, with infant-mother ratios at around 0.3–1.0 reported [25, 28–32]. Transient neonatal effects such as jitteriness, hypoglycemia, and respiratory disorders are described after intrauterine SSRI exposure [33, 34].

Designing a randomized controlled trial, we aimed to clarify several aspects of the effects of antidepressant treatment on the pregnant mother and her infant. In this pharmacokinetic part of the study, we aimed to clarify the variability in plasma sertraline concentrations during pregnancy and demonstrate whether TDM would improve the safety and efficacy of the treatment. In the infant, we aimed to measure the plasma levels of sertraline and relate these to the maternal concentrations and potential clinical symptoms in the new born.

## Materials and methods

### Clinical design and methods

This study is part of a double-blind randomized controlled trial examining the effects of sertraline and depression in pregnancy on the short- and long-term outcomes in the infants, the MAGDALENA study. The study design is fully described in the study protocol publication [35], and details on the recruitment, randomization, and follow-up are presented in the online supplement. All patients had an untreated moderate major depressive disorder at inclusion. Women with psychiatric or somatic comorbidities or any chronical drug treatment were excluded from the study. All included women were offered a 12-week program of Internet-based cognitive behavior therapy (I-CBT) with pregnancy-adapted treatment modules developed by our team [36]. Additionally, the women were either randomized to sertraline or placebo with the daily dose starting at one capsule á 25 mg. The treatment effect was followed up by the Montgomery Åsberg Depression Scale (MADRS) at the follow-up visits with the study midwife [37, 38]. The dose was increased in steps of one capsule when lacking treatment response, up to a dose of four capsules (100 mg of sertraline vs placebo). If patients in the placebo arm did not improve despite the I-CBT treatment, they were unblinded and switched to treatment with sertraline.

**Fig. 1:**
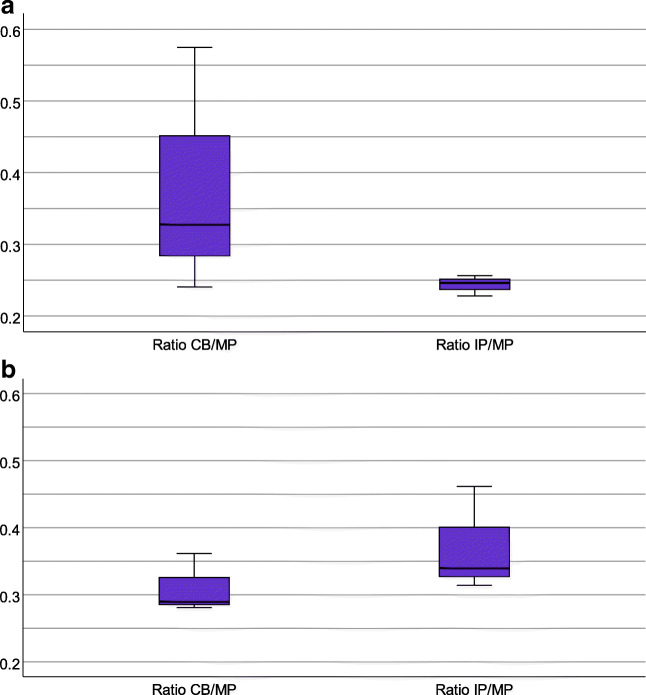
**a**, **b** Boxplots of the penetration ratios of sertraline (a) and desmethylsertraline (b). **a**) Penetration ratio of sertraline into cord blood (CB/MP, 5 mother-infant pairs): median 0.33; IQ-range 0.24–0.58; range: 0.14–1.17, and infant plasma (IP/MP, 5 mother-infant pairs): median 0.25; IQ-range 0.23–0.26; range 0.16–0.49. **b**) Penetration ratio of desmethylsertraline into cord blood (CB/MP, 5 mother-infant pairs): median 0.29; IQ-range 0.28–0.36; range: 0.24–1.42, and infant plasma (IP/MP, 5 mother-infant pairs); median 0.34, IQ-range 0.31–0.37; range 0.31–0.46

This pharmacology part of the study describes the sixteen included mother-infant-pairs, focusing on the nine mothers who in the end received treatment with sertraline (Suppl.Fig.1). Plasma sertraline and desmethylsertraline concentrations in the mothers were measured once in the second trimester around week 21 (range 17–25) of pregnancy and once in the third trimester around week 30 (range 26–36) of pregnancy, the morning after the delivery and 1-month postpartum. The infant concentrations are measured in cord blood and at 48 h of age together with the routine neonatal screening (Table 2, Fig. 1). All concentrations are steady-state total plasma concentrations. The babies were monitored with the modified Neonatal Abstinence Scale (NAS) according to Finnegan [39] during their first 48 h of life, and their plasma glucose concentrations (p-glucose) were measured at 6 and 48 h of age.

### Laboratory methods

Four milliliters of venous blood was collected from the women at each visit and from the umbilical cord at delivery into sodium heparin tubes. From the infants, 0.5 mL venous blood was collected at 48 h of age into lithium heparin capillary tubes. The drug analytical laboratory method and its performance are described in detail in the online supplement.

### Statistical analysis

Plasma concentrations originally presented in molar units (nmol/L) were converted to mass units (ng/mL) by multiplying the molar unit with the molecular weights of sertraline, 306.2 g/mol, and desmethylsertraline, 292.2 g/mol, to achieve the concentrations in ng/L and dividing by 1000 to achieve the concentrations in ng/mL [40]. A concentration-by-dose (C/D) ratio in (ng/mL)/(mg/day) was calculated by dividing the achieved concentration by the daily dose of sertraline in milligrams. Alteration ratios (AR) were used to analyze the difference between maternal plasma concentrations in the pregnant and the non-pregnant state, in accordance with previous studies [41]. Alteration ratios were calculated for the measuring points in pregnancy and at delivery by dividing these dose-adjusted concentrations with their non-pregnant reference, measured 1-month postpartum. Due to the nature of these analyses, the data were analyzed per protocol, and women were moved between treatment groups when treatment regime changed. To explore the foetal exposure, correlations was calculated between the concentration in maternal plasma and the ones in cord blood and infant plasma. The penetration ratio into cord blood (CB) and infant plasma (IP) was calculated by dividing the concentration in cord blood or infant plasma with the concentration in maternal plasma (MP), reflecting the penetration ratio into the infant.

Data is presented as medians, interquartile (IQ) ranges between the first(Q1) and the third(Q3) quartiles as well as ranges between min and max values. Correlations were computed to assess the relationship between the drug concentrations in maternal plasma and the infant. Because of the linearity of this correlation (Supp. Fig. 3), Pearson’s correlation coefficients were calculated. When searching for statistical significance in the decreased sertraline concentrations during pregnancy compared to postpartum, the non-parametric Wilcoxon’s signed rank test was used due to the small sample size. The non-parametric Spearman’s correlation test was used to study the correlation between the sertraline concentration and the treatment effect in the pregnant women (Section 2.2 of the online supplement). Statistical analyses were conducted with SPSS (version 26, IBM, Armonk, NY, USA).

## Results

### Patient characteristics

The study cohort consisted of 16 women recruited between May 2016 and March 2019. Ten women were randomized to sertraline and six to placebo treatment. In the end, nine women with two or more available sertraline plasma concentrations are included in the analysis (Table 2, Table 3, Supplemental Fig. 2). The randomization procedure and the flowchart of the study are described in detail in the online supplement (Supplemental Fig. 1). In two cases, infant drug concentrations are missing or lacking informed consent from the other parent, leading to seven mother-infant-pairs being analyzed for placental cross-over (Fig. 1, Supplemental Fig. 3).

Concomitant medications are presented in supplementary Table 1. The only concomitant psychotropic drug was promethazine, a first-generation antihistamine used temporarily by three women in the study. Temporary use of promethazine during pregnancy for pregnancy-related nausea and anxiety is common.

### Sertraline and desmethylsertraline plasma concentrations during pregnancy

The repeated concentration measures of sertraline and desmethylsertraline show an up to 10-fold interindividual variation (Table 2). The intraindividual dose-adjusted measures seem fairly consistent over the course of pregnancy with an increase postpartum, visualized in the line graph in supplemental Figure 2 (Table 2, Supplemental Fig. 2). The median dose-adjusted sertraline concentration increased with 67% from 0.15 to 0.25 (ng/mL)/(mg/day) from the second trimester to postpartum, but the variation was large, and the increase was not statistically significant (*p* = 0,345) (Table 2). The increase over time in the non-dose-adjusted concentrations might be related to the study design with increased dosage during pregnancy in most patients. We studied the correlation between plasma sertraline concentrations and treatment effect measured with a change in MADRS, without finding any significant correlations. This is further described in the online supplement, Section 2.2.

### Alteration ratios

We calculated alteration ratios between pregnant and non-pregnant state on the 8 patients with available non-pregnant reference concentrations measured 1-month postpartum. The median alteration ratio for sertraline during pregnancy is 0.83 and for desmethylsertraline 0.82, with an almost 10-fold variation in both (Table 3).

### Infant sertraline exposure

The median concentration of sertraline in maternal plasma at delivery (*n* = 8) was 14.38 ng/mL (range 3.64–24.17), and the median concentrations in the infants were 4.28 ng/mL (range 1.22–6.12) in cord blood (*n* = 5) and 4.59 ng/mL (range 1.25–7.04) in infant plasma at 48 h of age (*n* = 5). The median concentration of desmethylsertraline in maternal plasma at delivery (*n* = 8) was 33.60 ng/mL (range 7.01–31.36). Median concentrations in the infants were 9.93 ng/mL (range 4.96–17.23) in cord blood (*n* = 5) and 17.52 ng/mL (range 9.93–20.44) in infant plasma at 48 h of age (*n* = 5). We confirmed the concentrations being generally lower in the infants than in their mothers by calculating penetration ratios into cord blood (CB) and infant plasma (IP) (Fig. 1). Figure 1 also show a decreased ratio of sertraline and an increased ratio of desmethylsertraline between the samples measured from cord blood and in infant plasma at 48 h of age, indicating clearance of sertraline and conversion into desmethylsertraline within the first 48 h of life.

The correlations between the sertraline and desmethylsertraline concentrations in maternal plasma and the infant are fairly linear, as presented in supplemental Fig. 3. The Pearson correlation coefficients for these correlations were *r* = 0.70 (*p* = 0.19) for the correlation of sertraline concentration between maternal plasma and cord blood, *r* = 0.64 (*p* = 0.24) for sertraline concentration between maternal and infant plasma, *r* = 0.78 (*p* = 0.12) for desmethylsertraline concentration between maternal plasma and cord blood, and *r* = 0.83 (*p* = 0.08) for desmethylsertraline concentration between maternal and infant plasma.

### Neonatal outcomes

All infants are healthy at birth, with all 5' and 10' APGAR scores being 9 or 10 (Table 1). No baby showed any signs of severe withdrawal symptoms, classified as NAS > 8 points. One baby in the placebo group shows signs of moderate abstinence (NAS 7 points), and two babies in the sertraline treatment group show signs of mild abstinence (NAS 4 points), but none required any treatment for this (Table 1). One baby in the sertraline treatment group presented with a single low p-glucose of 2.5 mmol/L before the second feed but did not require any additional treatment apart from extra feeding. No other babies signs of hypoglycemia. One baby in the sertraline group was born preterm at 33 weeks of gestation and was the only baby who needed neonatal care. This baby did not have any complications from the premature birth. One baby in the placebo group was small for gestational age. All other babies were appropriate weight for gestational age. Two babies in the sertraline group and one in the placebo group experienced mild respiratory disorders with no need of neonatal care or respiratory support with continuous positive airway pressure (CPAP) after the initial 20 min in life. One baby in the sertraline group experienced transient jitteriness at 1 week of age with a normal EEG at check-up.

**Table 1:** Patient characteristics and delivery outcomes

Patient	Assignedtreatment	Maximal dose of Sertraline (mg)	Maternal age (years)	GA (w+d)	Delivery mode	Infant sex	Infant weight (g)	Weight percentile	APGAR	NAS
1	Sertraline	50	26	33 + 2	Vaginal	Boy	2021	43	9.9.10	4
2	Sertraline	50	31	39 + 4	Vaginal instrumental	Girl	3510	59	9.10.10	2
3	Sertraline ^a^	50	31	38 + 5	Vaginal	Boy	3420	50	10.10.10	NA
4	Sertraline	50	32	39 + 3	Vaginal	Girl	3610	68	9.10.10	4
5	Sertraline	100	32	37 + 0	Vaginal	Boy	3400	74	9.10.10	NA
6	Sertraline	75	32	37 + 3	Vaginal	Boy	3330	62	7.9.9	3
7	Sertraline	75	32	37 + 6	Planned CS	Girl	2890	32	9.10.10	0
8	Sertraline	75	32	39 + 0	Planned CS	Boy	3510	53	9.10.10	2
9	Sertraline ^d^	75	36	38 + 3	Vaginal	NA	NA	NA	NA	NA
10	Sertraline ^b^	75	39	39 + 2	Vaginal	Boy	3150	24	6.9.10	NA
11	Placebo	0	24	38 + 4	Vaginal	Girl	3300	52	9.10.10	NA
12	Placebo ^c^	75	28	37 + 4	Vaginal	Girl	3140	55	7.9.10	2
13	Placebo	0	29	42 + 3	Vaginal	Girl	3380	38	9.10.10	7
14	Placebo	0	33	39 + 5	Vaginal	Girl	2732	6	9.10.10	0

*GA* gestational age at delivery in weeks + days. *CS* cesarean section. *NAS* modified neonatal abstinence scale according to Finnegan in Swedish [39]. Data of all included patients apart from the two early dropouts from the placebo group is presented. *NA* not available

^a^Treatment discontinued in 2nd trimester. ^b^Treatment discontinued in 3rd trimester. ^c^Randomized to placebo but active treatment from 2nd trimester. ^d^Lacking informed consent for infant data

## Discussion

This study on sertraline concentrations in pregnant women and their infants demonstrates an over 10-fold interindividual variability in sertraline and its metabolite plasma concentrations across the course of the pregnancy (Table 2), supporting previous studies from both pregnant and non-pregnant populations [7, 12, 17]. The intraindividual variability during pregnancy is low, presented in supplemental Fig. 2. We found an overrepresentation of lower concentrations, with only a few notably higher concentrations as reported in non-pregnant populations, adding to the varying results from studies in pregnant women [7, 12, 17, 42]. This variability is probably due to genetic differences in drug metabolic capacity, mainly caused by the polymorphism of the CYP2C19-enzyme [22, 26]. We also observe an up to 10-fold variation in the alteration ratios between pregnant and non-pregnant state, with the median ratio just below 1 (Table 3). This supports the findings of a recent study and suggests a genetic variation in the pregnancy-induced changes of the drug metabolizing enzymes as well [41]. However, it might also be explained by altered levels of protein binding during pregnancy [21]. The median drug-adjusted sertraline plasma concentration increased by 67% from the second trimester to postpartum (Table 2, Supplemental Fig. 2). However, probably due to sample size, this was not statistically significant. Based on these findings, together with results from previous studies, it seems wise to monitor plasma sertraline concentrations during and after pregnancy [7, 12, 25, 43].

**Table 2:** Sertraline and desmethylsertraline plasma concentrations

	**N**	Dose (mg)	Measured concentration (ng/mL)		Dose-adjusted concentration (ng/mL)/(mg/day)	
		Median (range)	Median (Q1–Q3)	Range	Median (Q1–Q3)	Range
Sertraline						
2nd trimester	7	50 (50–50)	7.65 (5.81–12.09)	3.98–17.14	0.15 (0.12–0.24)	0.08–0.34
3rd trimester	9	50 (50–75)	9.49 (7.34–17.14)	1.53–20.81	0.19 (0.12–0.23)	0.03–0.38
At delivery	8	75 (50–100)	14.38 (7.65–18.67)	3.64–24.17	0.19 (0.15–0.25)	0.07–0.32
Postpartum	8	75 (50–75)	17.9 (8.87–19.35)	6.12–52.02	0.25 (0.17–0.29)	0.12–0.69
Desmethylsertraline						
2nd trimester	7	50 (50–50)	24.25 (22.65–32.29)	15.78–10.52	0.49 (0.45–0.65)	0.32–0.88
3rd trimester	9	50 (50–75)	35.06 (28.05–55.52)	10.52–61.36	0.70 (0.47–0.74)	0.21–1.11
At delivery	8	75 (50–100)	33.60 (22.95–46.75)	7.01–61.36	0.46 (0.37–0.62)	0.14–0.82
Postpartum	6	75 (50–75)	45.29 (28.56–78.89)	16.95–87.66	0.69 (0.43–1.05)	0.34–1.17

Sertraline dose presented as median and range (min-max) and measured and dose-adjusted sertraline and desmethylsertraline plasma concentrations presented as medians, interquartile ranges (Q1–Q3) and ranges (min-max)

**Table 3:** Alteration ratios

Alteration ratio, median (min-max)	2nd trimester	3rd trimester	Delivery
Sertraline	0.88 (0.49–1.37)	0.69 (0.17–1.23)	0.85 (0.21–2.08)
Desmethylsertraline	0.94 (0.47–1.38)	0.89 (0.62–1.44)	0.72 (0.25–1.22)

Alteration ratios between pregnant and the non-pregnant state measured 1-month postpartum for sertraline and desmethylsertraline, presented as a median (min-max) of the 8 women with sufficient data

Median plasma concentrations of sertraline in the infants were found to be around a third of the mother’s, suggesting a low placental passage of sertraline. This is in line with findings from a handful earlier studies and is reassuring regarding the safety of sertraline during pregnancy [29–32]. We also found a linearity in this correlation not previously described, presented in the online supplement. The low infant levels might be explained by sertraline mostly being bound to AAP, present in the infant at levels around a third of the mother’s. The very high proportion of protein-bound sertraline and its metabolite probably also explains why the sertraline concentration in the infant is low even though the placental permeability for the free form of the drug is markedly increased at the end of the pregnancy. Our results also suggest that in the infant, sertraline is effectively cleared and converted to desmethylsertraline (Fig. 1), which is in line with one previous study showing a total clearance of sertraline at five days of age [31].

Previous studies have shown that both maternal major depression and exposure to SSRIs are associated to increased risks for intrauterine growth restriction, prematurity, and neonatal adaptation problems including jitteriness, respiratory problems, and hypoglycemia [30, 33, 44]. In the present study, none of the nine exposed infants had any signs of asphyxia, intrauterine growth restriction, or poor neonatal adaptation. One infant exposed to sertraline experienced transient jitteriness at 1 week of age and two infants in the sertraline group and one in the placebo group experienced a transient need of respiratory support with CPAP, for less than 20 min. Group sizes were too small to determine whether any of these outcomes are significant. Large postpartum hemorrhage when using SSRI is a known complication [45, 46], and in this small sample, we found a tendency towards increased postpartum hemorrhage in the women treated with sertraline, presented in Section 2.5 of the online supplement.

The main strength of our study is that the indication for the treatment was well-based by the study psychiatrist and same in all patients, which is often not the case in naturalistic studies. As per protocol, the patients also did not have any significant concomitant medications. The greatest limitation of our study turned out to be the sample size, and none of the results is statistically significant. However, previous studies have encountered the same limitation, and increasing the sample sizes to achieve significant results is our common challenge.

As sertraline is highly protein bound, it would have been of great interest to also measure the concentrations of the free drug. This would have had potential to increase our knowledge about the pregnancy changes of sertraline concentrations and the transfer of sertraline to the infant.

Considering SSRIs being such a common treatment during pregnancy, it is a shame that studying its effects is so complicated, with small sample sizes limiting clinical studies and register-based studies struggling with confounding, especially confounding by the severity of the underlying disease. In the online supplement, we are discussing the challenges of RCT:s in this field. We hope that our finding of infant plasma concentrations being just a third of the maternal concentration can bring further assurance to the safety of the use of sertraline during pregnancy. It is also assuring for the safety of the treatment that the repeated measures of maternal concentrations are reasonably stable across the course of the pregnancy.

## Conclusions

In our small cohort, sertraline concentrations during pregnancy do seem lower than the ones postpartum, probably indicating an increased metabolism during pregnancy. However, this is not true for all of the women, supporting the role of genetic differences. Therapeutic drug monitoring during pregnancy can increase the safety and efficacy of the treatment on group level, finding the poor metabolizers at risk for elevated drug concentrations with potential to cause adverse effects, as well as the women with low concentrations as a reason to lack of treatment effect. In our study, the infant plasma concentrations were low, and no serious adverse events were found in the included infants. Therefore, based on our material together with previous studies in the field covering the long-term effects [5, 6], it seems as if sertraline is safe to use for moderate depression during pregnancy when indicated. Further, collecting novel un-confounded data through randomized trials in this field is very difficult, why register based studies have an important role in future.

## Supplementary Information

ESM 1 (PDF 730 kb)
