# Supplementary material for: Sertraline concentrations in pregnant women are steady and the drug transfer to their infants is low
Source: Eur J Clin Pharmacol. 2021 Mar 22;77(9):1323–31. doi: 10.1007/s00228-021-03122-z (PMC8346399; doi:10.1007/s00228-021-03122-z)
Supplement: Supplementary file 1 — (PDF 730 kb) [file 228_2021_3122_MOESM1_ESM.pdf]

Online Supplement for:

Sertraline concentrations in pregnant women are steady and the drug transfer to their infants is low

Published in European Journal of Clinical Pharmacology on March 22nd, 2021.

Heinonen E[1,2], Blennow M[1,2], Blomdahl-Wetterholm M[3], Hovstadius M[1], Nasiell J [4,5], Pohanka A[6,7], Gustafsson LL[6,7] , Wide K[1,8]

Division of Paediatrics, Department of Clinical Science, Intervention and Technology (CLINTEC), Karolinska Institutet, Stockholm[1], *Department of Paediatrics and Newborn medicine at Karolinska University Hospital, Stockholm[2], Psychiatry South West, Stockholm Healthcare Region[3], Department of Clinical Sciences, Danderyd Hospital, Karolinska Institutet, Stockholm[4], Department of Obstetrics and Gynaecology at Danderyd Hospital, Stockholm[5],* Division of Clinical Pharmacology, Department of Laboratory Medicine, Karolinska Institutet, Stockholm[6], Department of Clinical Pharmacology, Karolinska University Hospital, Stockholm[7],*Department of Paediatrics and Emergency Paediatrics at Karolinska University Hospital, Stockholm, Sweden.[8]*

Corresponding author: Essi Heinonen, *essi.heinonen@ki.se*

This supplement presents method descriptions, further graphs for better visualization of the results and a discussion about randomization in the study.

1. Supplemental method descriptions

1.1 Supplemental information on study population and design

The study was unique in its randomized controlled design comparing the fetal effects of exposure to maternal depression with and without exposure to sertraline, aiming to separate the effects of the treatment from the effects of the underlying disease on the infants. The patients were recruited between February 2016 and August 2019. The recruitment was terminated early due to slow recruitment pace. We are therefore lacking the power to answer the primary question about the neurodevelopmental effects on the infant. However, the collected plasma drug concentrations in the mothers treated with sertraline and their infants are of interest and are therefore published. The study was a collaboration between the Departments of Psychiatry Southwest, Gynecology and Obstetrics, Pediatrics and Clinical Pharmacology at the Karolinska University Hospital and their counterparts at the Department of Clinical Intervention and Technology at Karolinska Institute.

Women in early pregnancy were recruited across Stockholm Healthcare Region with 2.3 million inhabitants and 29 000 deliveries annually, through antenatal clinics, social media, and advertisements in conventional media.[1] A first screening was made with Edinburgh Postnatal Depression Scale (EPDS) with a cut-off of 13 points for inclusion.[2] The subjects scoring above this

cut-off and lacking exclusion criteria such as severe psychiatric or somatic disease or ongoing chronic medication were scheduled to meet one of the study psychiatrists for a clinical evaluation, including a SCID-I directed interview (Structured Clinical Interview for DSM IV axis I disorders),[3] final review of eligibility criteria and signing of the final informed consent form. As a result of this rigorous inclusion procedure, all included women fulfilled the criteria of untreated moderate major depressive disorder in early pregnancy, without any psychiatric or physical comorbidities or chronical treatment.

1.2 Laboratory methods

4mL venous blood was collected from the women at each visit and from the umbilical cord at delivery into Sodium Heparin tubes. From the infants, 0.5mL venous blood was collected at 48 hours of age into Lithium Heparin capillary tubes. The samples were transported to the laboratory at room temperature the same day, or when collected during the weekend, the next following weekday. The samples were centrifuged at arrival at the laboratory and plasma was stored at -70°C for later joint analysis of all collected samples. 0.1ml of plasma was needed for each analysis. Total plasma concentrations of sertraline and desmethylsertraline were determined by Liquid chromatography coupled to tandem mass spectrometry (LC-MS/MS). The sample work-up was based on protein precipitation by MeOH containing isotopically labeled internal standards. After vortexing and centrifugation, the supernatant evaporated to dryness and then reconstituted with 0.1% aqueous formic acid. An aliquot was then injected into the analytical system. The method was validated according to the European Medicines Agency's Guideline on bioanalytical method validation. The quantification range was 1-500 nmol/L and 5-850 nmol/L for sertraline and desmethylsertraline, respectively. The total coefficient of variation was 6.8% at the lower limit of quantification (LLOQ) and ≤3.1% in the quantification range for sertraline and 5.6% and ≤3.5% for desmethylsertraline. Accuracy was -4% at LLOQ and between +5.3 to +12,3% in the quantification range for sertraline and +11.7% at LLOQ and between -10.2% to -1.8% for desmethylsertraline.

2. Supplemental results

2.1 Recruitment of the randomized trial

Supplemental figure 1 describes the randomization of the included patients and the extent of succeeded follow-up. Two women randomized to sertraline discontinued drug therapy, in the second and third trimester, respectively. In two cases, infant drug concentrations were missing or lacking informed consent from the other parent. Two patients with placebo treatment are considered drop-outs with no further analysis as they discontinued their treatment before the first concentrations were taken. A third patient in the placebo treatment arm did not reach treatment effect, why the randomization code was broken, and active treatment started 4 weeks after inclusion. Therefore, only three patients fulfilled the study within the placebo treatment group.

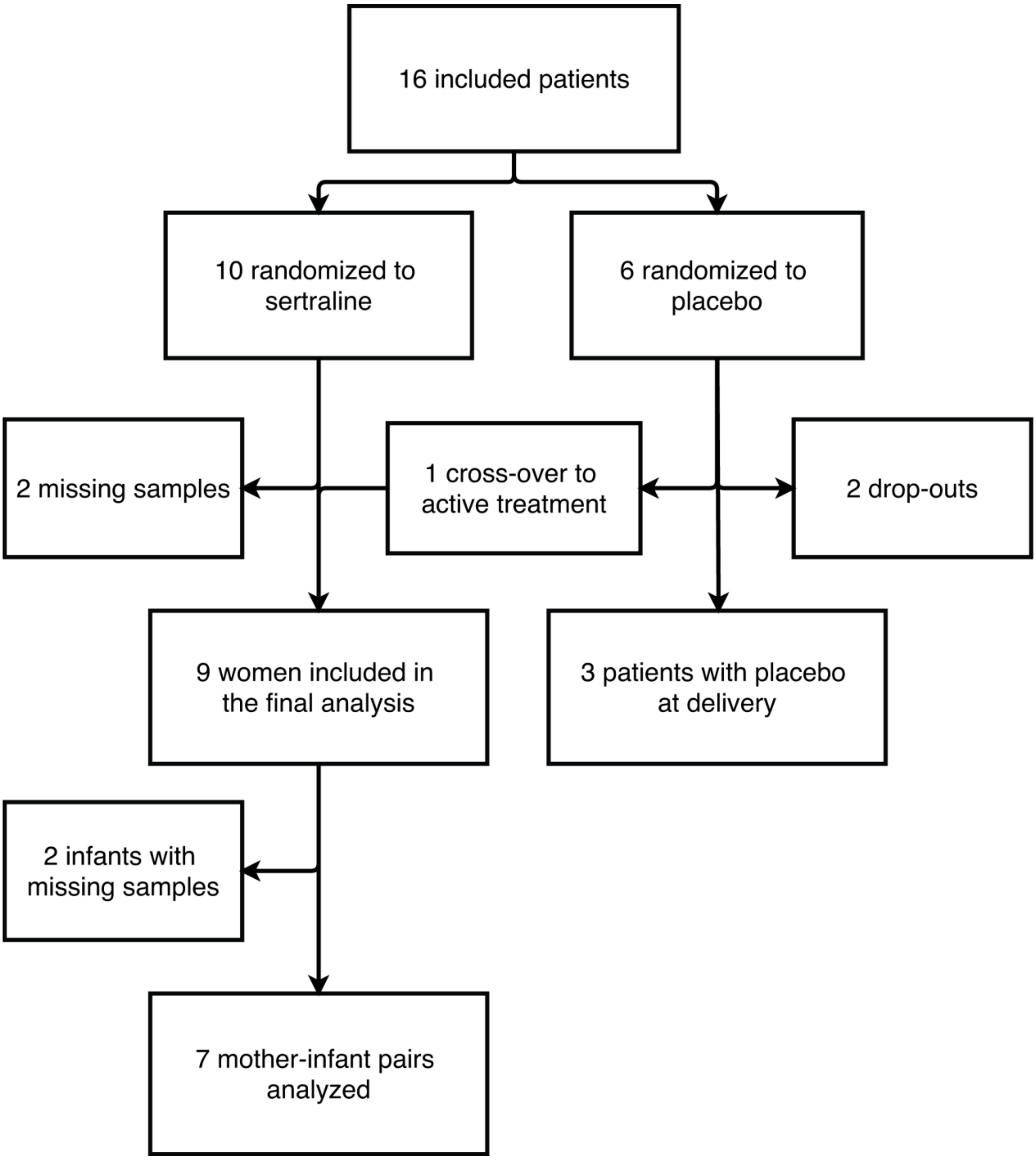

Suppl. Fig.1 Flow-chart of the recruited patients. Nine women with two or more available drug concentrations were included in the maternal analysis, and seven mother-infant-pairs had sufficient data for analysis of placental cross-over. Three patients in the placebo group fulfilled the study but they were not analyzed in this pharmacological part of the study.

2.2 Treatment evaluation

At inclusion, all 16 participants were depressed according to psychiatrist evaluation including Structured Clinical Interview for DSM IV axis I disorders (SCID-I) and MADRS. 14 women had over 21 points on MADRS, the threshold of a high probability of having a major depressive disorder (MDD), and two women 20 points. Generally, the MADRS scores decreased during the course of the

treatment. At the end of the study one month postpartum ten women were non-depressed according to MADRS (<12 points). Three patients in the sertraline treatment group (two of whom lacked all plasma concentrations) and one in the placebo group had elevated MADRS scores and were referred to further treatment as usual after a clinical evaluation by the study psychiatrist. The two drop-outs in the placebo group are lacking MADRS scores postpartum. We studied the correlation between the sertraline concentration and treatment effect measured by change in MADRS scores, without any correlations found or even suspected, p-values ranging between 0,38-0,81 for the different time points.

2.3 Concomitant medications

An on-going chronical medication for a psychiatric or a somatic chronic disorder was an exclusion criteria for this study. A few medications, with temporary usage or with local administrations were approved. The full list of concomitant medications is found in Supplemental table 1.

Patient	Assigned treatment	Concomitant medication
1	Sertraline	
2	Sertraline	Antihistamines, Fluconazole (locally), Metronidazole, Promethazine/Ephedrine/Caffeine
3	Sertraline	
4	Sertraline	
5	Sertraline	
6	Sertraline	Levothyroxine, Salbutamol, Fluticasone/Salmeterol
7	Sertraline	Metoclopramide, Promethazine
8	Sertraline	Promethazine
9	Sertraline	Acetylic Salicylic Acid
10	Sertraline	Levothyroxine, Promethazine/Ephedrine/Caffeine
11	Placebo	
12	Placebo	
13	Placebo	
14	Placebo	Paracetamol/Codeine

Suppl. Table 1. Concomitant medications.

2.3 Sertraline plasma concentrations during pregnancy and postpartum

Table 2 in the main publication presents the medians, ranges and interquartile ranges for the sertraline concentrations in the included women. The graphs in supplemental figure 2 show visually the intraindividual consistency and lack of intraindividual variation in plasma sertraline and desmethylsertraline concentrations during pregnancy.

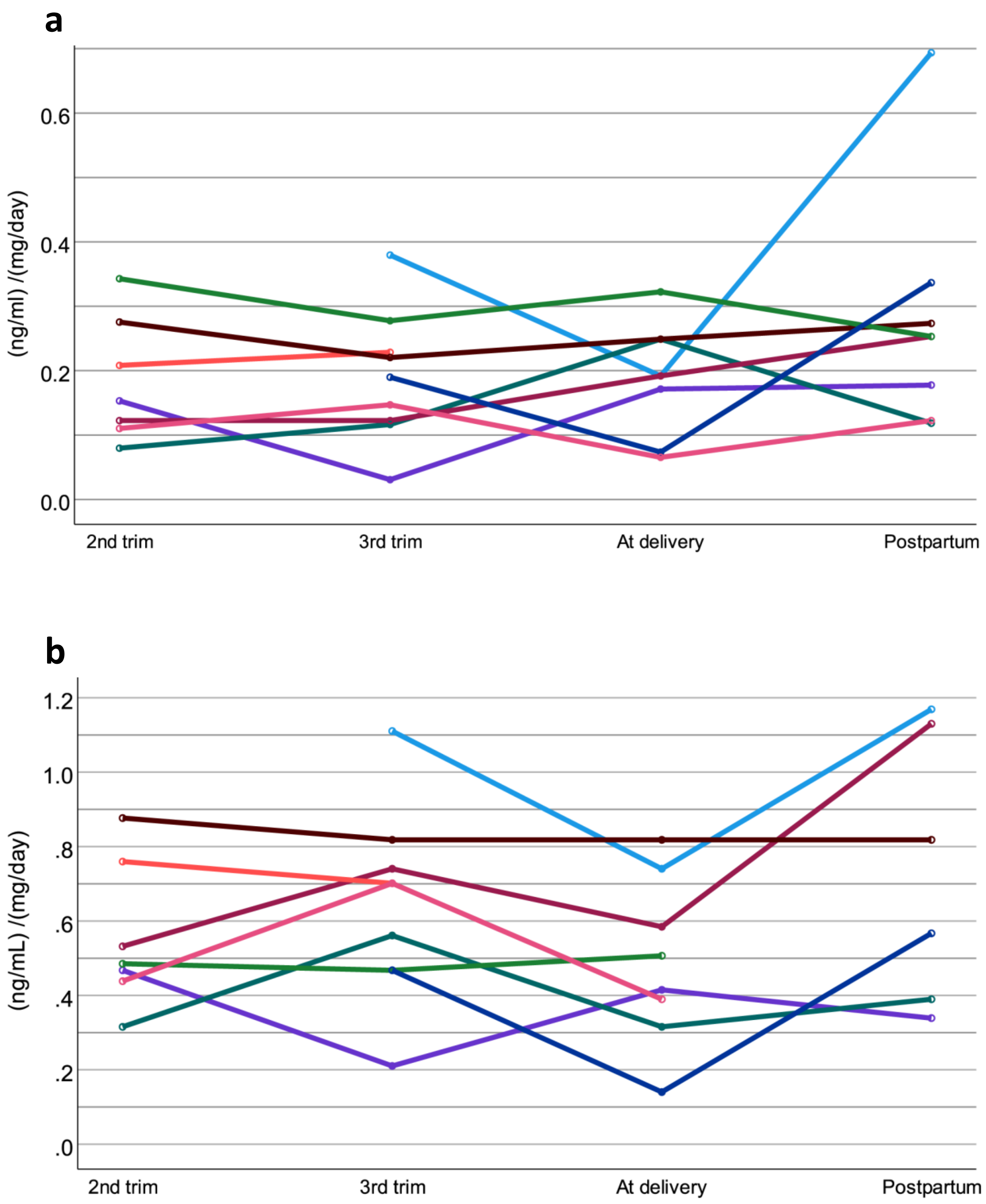

Suppl.Fig.2a,b TDM concentrations of Sertraline (a) and Desmethylsertraline (b) during pregnancy. Each line represents a study participant. The concentrations were measured at visits in the second trimester (around pregnancy week 21), in the third trimester (around pregnancy week 30), at delivery and one month postpartum. All concentrations are dose-adjusted

2.4 Correlation between the maternal and infant plasma concentrations

The graphs in supplemental figure 3 visually show the correlation between maternal plasma and infant levels measured in both cord blood and infant plasma at 48 hours of age, described in chapter 3.4 in the main manuscript.

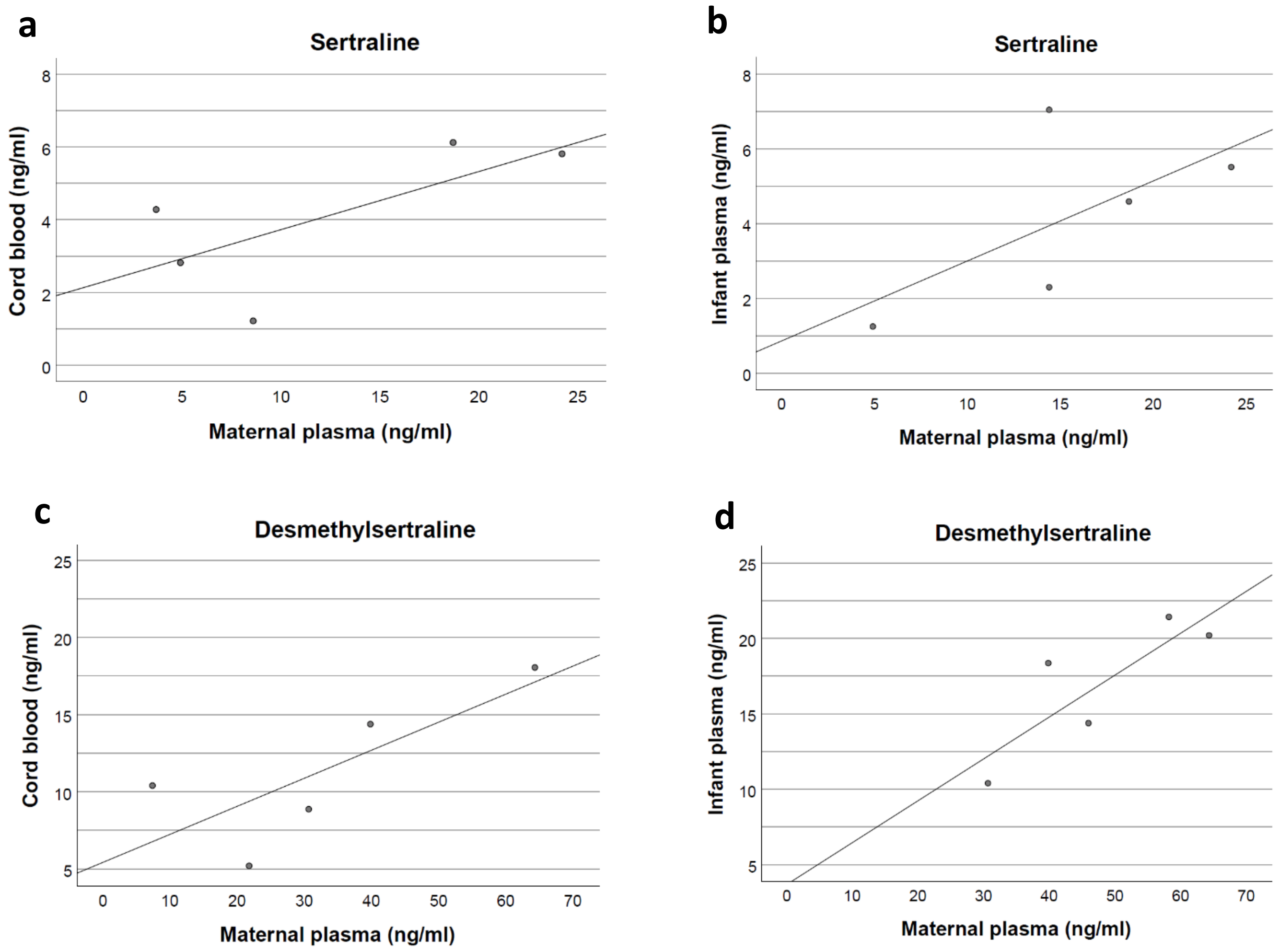

Supp.Fig.3a-d Scatter plot of the correlations for sertraline (a,b) and desmethylsertraline (c,d) between the mother and the infant. Sertraline concentrations in a) maternal plasma vs cord blood (r^2 linear=0,49) and b) maternal vs infant plasma (r^2 linear =0,41). Desmethylsertraline concentrations in c) maternal plasma and cord blood (r^2 linear =0,61) and d) maternal and infant plasma (r^2 linear =0,70)

2.5 Explorative outcomes - Peripartal hemorrhage

Two women in the sertraline group had a severe postpartum hemorrhage (PPH) with over 2000mL blood loss, one of them after instrumental delivery, one after vaginal delivery. One woman in the placebo group had a moderate PPH with a 1000-2000mL blood loss after a vaginal delivery. All other women had a PPH of less than 1000mL.

3. Supplemental discussion on recruitment

Our original randomization was skewed, with ten women randomized to sertraline and six to placebo treatment, but as we followed double-blinded block randomization this skewness should be due to chance and not affected by any systemic bias. Adding to the skewness, we had a 50% drop out in the placebo group, which might be a sign of lack of treatment effect. The patients in the placebo group were treated with, apart from the placebo capsules, an internet-based cognitive behavior therapy specially customized for depression during pregnancy and tested in a pilot study to have a good compliance and effect.[4] The compliance to the therapy in our study was generally lower than in the pilot study with only three out of ten modules filled by the patients on average, when five completed modules are seen as good compliance.[4]

After failing to complete the recruitment for a complete and more substantial RCT on both short-term and long-term effects of SSRI-exposure during pregnancy, we cannot conclude if there are any later neurodevelopmental effects. We also know that a similar effort in the Netherlands faced the same challenges with recruitment.[5] Therefore we have respect for all the efforts put in clinical studies in this challenging and yet so important field of research. Further discussions should be held in different forums how to proceed with better safety data on especially long-term effects on the infants.

4. Supplemental references

1. Socialstyrelsen. Statistics database for pregnancys dan. https://sdb.socialstyrelsen.se/if_mfr_004/resultat.aspx. Accessed 10/14 2020
2. Hellden A, Madadi P (2013) Pregnancy and pharmacogenomics in the context of drug metabolism and response. Pharmacogenomics 14 (14):1779-1791. doi:10.2217/pgs.13.176
3. Kübler U (2013) Structured Clinical Interview for DSM-IV (SCID). In: Gellman MD, Turner JR (eds) Encyclopedia of Behavioral Medicine. Springer New York, New York, NY, pp 1919-1920. doi:10.1007/978-1-4419-1005-9_66
4. Forsell E, Bendix M, Hollandare F, Szymanska von Schultz B, Nasiell J, Blomdahl-Wetterholm M, Eriksson C, Kvarned S, Lindau van der Linden J, Soderberg E, Jokinen J, Wide K, Kaldo V (2017) Internet delivered cognitive behavior therapy for antenatal depression: A randomised controlled trial. J Affect Disord 221:56-64. doi:10.1016/j.jad.2017.06.013
5. Molenaar NM, Brouwer ME, Bockting CL, Bonsel GJ, van der Veere CN, Torij HW, Hoogendijk WJ, Duvekot JJ, Burger H, Lambregtse-van den Berg MP (2016) Stop or go? Preventive cognitive therapy with guided tapering of antidepressants during pregnancy: study protocol of a pragmatic multicentre non-inferiority randomized controlled trial. BMC Psychiatry 16:72. doi:10.1186/s12888-016-0752-6
